# Enhancing Kidney Vasculature in Tissue Engineering—Current Trends and Approaches: A Review

**DOI:** 10.3390/biomimetics6020040

**Published:** 2021-06-16

**Authors:** Charlotta G. Lebedenko, Ipsita A. Banerjee

**Affiliations:** Department of Chemistry, Fordham University, 441 East Fordham Road, Bronx, NY 10458, USA; clebedenko@fordham.edu

**Keywords:** renal tissue engineering, vascularization, organoids, decellularized matrices

## Abstract

Chronic kidney diseases are a leading cause of fatalities around the world. As the most sought-after organ for transplantation, the kidney is of immense importance in the field of tissue engineering. The primary obstacle to the development of clinically relevant tissue engineered kidneys is precise vascularization due to the organ’s large size and complexity. Current attempts at whole-kidney tissue engineering include the repopulation of decellularized kidney extracellular matrices or vascular corrosion casts, but these approaches do not eliminate the need for a donor organ. Stem cell-based approaches, such as kidney organoids vascularized in microphysiological systems, aim to construct a kidney without the need for organ donation. These organ-on-a-chip models show complex, functioning kidney structures, albeit at a small scale. Novel methodologies for developing engineered scaffolds will allow for improved differentiation of kidney stem cells and organoids into larger kidney grafts with clinical applications. While currently, kidney tissue engineering remains mostly limited to individual renal structures or small organoids, further developments in vascularization techniques, with technologies such as organoids in microfluidic systems, could potentially open doors for a large-scale growth of whole engineered kidneys for transplantation.

## 1. Introduction

As the gap between organ need and donor availability continues to widen, the need for alternative methods of tissue replacement or regeneration rises [1]. In addition to donor shortage, surgical transplant methods present significant limitations due to immunological rejection, post-transplant infection, and possible harm to the donor [2]. To circumvent these drawbacks, scientists have been developing new regenerative medicine methodologies such as tissue engineering (TE). The goal of TE is to create tissue replacements derived from the patient’s autologous cells and engineered biomimetic scaffolds that can be grown in vitro and implanted to promote in vivo regeneration of the original tissue [3]. TE requires the proper choice of the scaffold biomaterial, appropriate cell types, an assortment of growth factors, and biomimetic components that will encourage development and organogenesis. The ideal TE scaffold should successfully mimic the target organ’s natural extracellular matrix (ECM) by promoting cell attachment, migration, proliferation, differentiation, tissue vascularization, and the flow of nutrients and waste [4]. Furthermore, a biodegradable scaffold that breaks down into harmless byproducts as the engineered tissue replaces it with its own secreted ECM is needed to avoid future scaffold removal through additional surgeries, formation of bacterial colonization, and other immunological reactions [5,6]. Other vital features of the scaffold include ease of synthesis, tissue-specific mechanical properties, and a diminished immune response [7]. 

The field of TE has seen major advances in engineering organs such as skin, cartilage, cornea, bladder, and bone, but human applications remain limited due to high costs and the failure of grafts to thrive in vivo [3]. While the engineering of relatively simple, avascular tissue, such as skin and cartilage, has seen great success in TE, the engineering of larger organs with intricate structures and vascular networks, such as the kidney, has seen limitations [8]. A major difference is that avascular tissues do not require extensive angiogenesis or vasculogenesis [9]. A majority of blood vessels within the peripheral, cerebral, cardiac, and renal vasculature are smaller in diameter (lower than 6 mm), and blood vessels in those organs have been shown to be more difficult to replace with synthetic polymer grafts due to a relatively inadequate match in mechanical properties, thrombus formation, and hyperplasia in the innermost membranes [10,11]. Without vascularization, the growth of any larger organ is impossible due to a lack of oxygen and nutrient delivery to the inside of the tissue causing graft necrosis [12]. The upper limit for avascular tissue is around 400 μm, so any tissue with a dimension larger than this will require almost immediate vascularization to prevent hypoxic conditions from killing inner cells [13]. Lack of proper vascularization both before and after implantation is one of the greatest obstacles to engineering large tissues, so the development of vascularization techniques for TE is of the utmost importance.

Chronic kidney disease causes over half a million patients to reach end-stage renal disease each year, and the only viable treatment options are dialysis and transplant, where the short half-life of the kidney often makes a second transplant necessary [14]. Consequently, the kidney is the single most sought after organ for transplantation [15]. The kidney has presented challenges because of its highly complex 3D structure, with specialized compartments, significant vascularization, and a diverse array of cell types and physiological functions [16]. Since nephrons cannot regenerate on their own once damaged [17], the need to engineer a proper scaffold with sufficient stability, porosity, and biocompatibility that promotes kidney cell differentiation, nephrogenesis, and organ vascularization remains high [18]. This review delves into some of the biological cues with regard to renal structure and current methodologies including a comparison of top-down versus bottom-up approaches being explored for improving vasculature in kidney TE.

## 2. Renal Vascular Structure and Cellular Composition 

**Figure 1:**
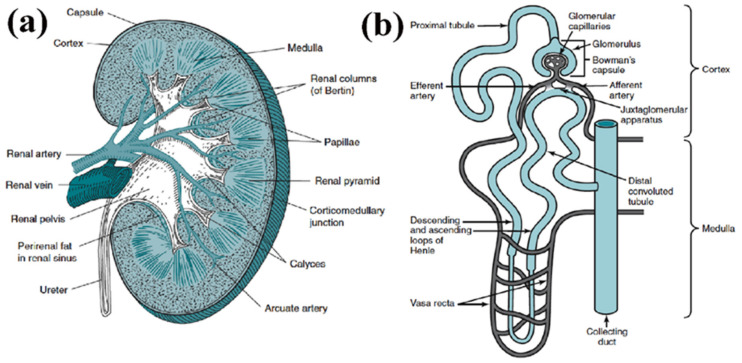
(**a**) Structure of kidney, comprised of renal cortex, medulla and pelvis. (**b**) Structure of an individual nephron, which comprises the renal corpuscle (glomerulus and Bowman’s capsule) and the renal tubule. (Reproduced with permission from Elsevier, adapted from reference [25].).

The major structural and functional unit of the kidney is the nephron, whose components include the glomeruli, renal tubules, and Bowman’s capsule (Figure 1), all significantly different in their ECM and cellular compositions [19,20]. Mimicking the natural ECM when designing scaffolds is thus particularly difficult for kidney TE due to the diverse ECM compositions. Furthermore, the kidney consists of over twenty types of epithelial cells, vascular cells, mesangial cells, and interstitial cells, all of which are essential for proper kidney functioning [21]. One of the most difficult components to replicate through TE approaches is the kidney vasculature. Human kidneys receive between 20 and 25% of cardiac output, so the kidney vasculature must be able to withstand significant blood flow and support proper glomerular filtration [22]. Cells of the renal vasculature include endothelial cells, vascular smooth muscle cells, pericytes, mesangial cells, and fibroblasts [23]. Kidney capillaries are comprised of endothelial cells covered by pericytes which are key to angiogenesis and vascular survival [24]. Overall, the kidney vasculature is extremely complex, making it one of the greatest challenges to overcome in TE approaches. 

The development of kidney vasculature entails vasculogenesis and angiogenesis. Vasculogenesis, or the de novo formation of blood vessels from endothelial progenitor cells, is active in early development of the kidney, while angiogenesis, or the outgrowth and remodeling of new blood vessels from existing ones, is active at a later developmental stage. Several studies have provided detailed reports of the development of kidney vasculature. Both angiogenic hemangioblast precursor cells from the embryo as well as vasculogenic endothelial precursors within the embryonic kidney itself are required for the development of kidney vasculature, suggesting that an artificially grown, in vitro, kidney will require factors from the surrounding environment to induce vascularization [26].

Some of the important considerations for vascularization in engineered tissues are the type of endothelial cells (ECs) used and their ratio to tissue-specific stem cells or other cell types [27,28]. For example, the co-culture of induced pluripotent stem (iPS) cell-derived ECs with mural cells such as pericytes formed human blood vessel organoids [29]. Similarly, mesenchymal stem cells enhanced the vasculogenic and pro-angiogenic activities of endothelial colony forming cells [30]. Additionally, M0 and M2 macrophages have been used in combination with ECs to enhance vascularization in a 3D hydrogel scaffold [31]. Endothelial progenitor cells (EPCs) have been used as an alternative as they are relatively easier to gather from the patient’s blood or bone marrow than organ-specific ECs [32]. In addition to the types of cells, the proangiogenic growth factors and the gradients in which they are present are essential for proper vasculature development [33]. The depth of tissue invasion by vasculature and the density of blood vessels formed are closely related to growth factor gradient magnitude [34].

## 3. Whole-Kidney Tissue Engineering—The Top-Down Approach

Approaches to kidney TE which utilize the entire native structure of the kidney and therefore require a donor organ include the use of decellularized ECM or vascular corrosion casts. These can be thought of as top-down approaches since the original donor organ is used to make a scaffold that preserves the original structural and mechanical properties of the organ and can then be repopulated with autologous cells for regenerative medicine applications. 

### 3.1. Decellularized ECM

The repopulation of decellularized kidney scaffolds with autologous cells holds promise as a method for whole-kidney TE. Many in-depth articles have been published on the use of decellularized ECM (dECM) [35]. Several decellularization methods that leave whole intact renal scaffolds with minimal damage to ECM mechanical integrity and biological activity have been described [36,37]. However, there is still no consensus on a standardized decellularization procedure. The main challenges for dECM include preserving the greatest amount of tubular structure and vasculature in dECMs, determining the appropriate cell types and culture systems for recellularization, and defining the appropriate maturation point that the graft can be transplanted into the patient. It has been shown that dECM maintains glomerular morphometry, vascular resilience, and critical growth factors [38]. While scientists have yet to successfully repopulate the entire scaffold with proper cell differentiation, major strides have been made towards the eventual clinical application of kidney dECMs. For example, rat kidney dECM seeded with epithelial and endothelial cells perfused in a whole-organ bioreactor produced rudimentary urine in vitro [39]. These grafts transplanted in rats were perfused by the host vasculature and produced urine in vivo. This section will focus on the decellularization and recellularization techniques for recreating kidney vasculature. 

**Figure 2:**
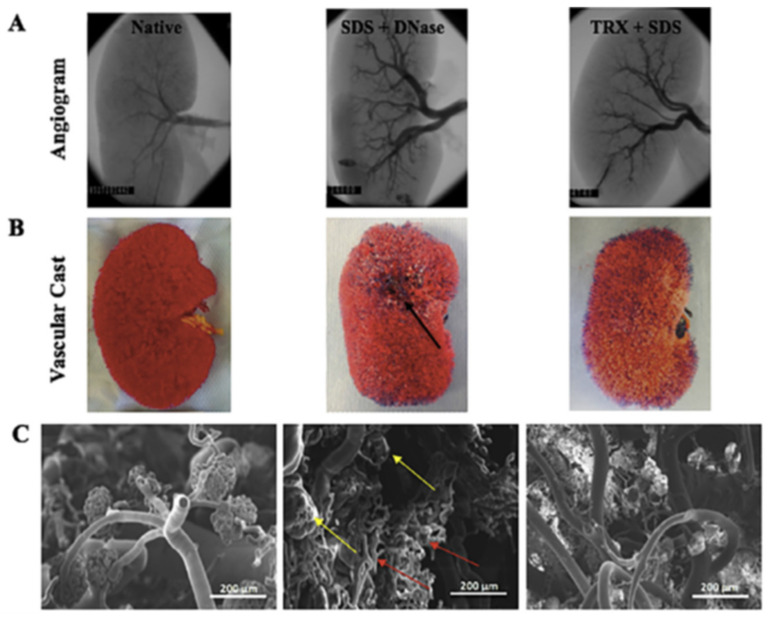
Comparison of vascular microarchitecture of native versus decellularized kidney scaffolds formed as a result of SDS and DNASE and triton X and SDS treatment. (**A**) Fluoroscopic angiogram images, (**B**) vascular casting, and (**C**) SEM images of the vascular cast (50 and 150 × magnification). Defect areas (black arrow) in vascular casts and damaged glomerular structures (yellow arrows) and blood vessels (red arrows) are shown as a result of DNASE and SDS treatment. (Reproduced with permission from Elsevier, adapted from reference [41].)

In order to optimize the scaffold’s vascular integrity, the method used for decellularization of kidney tissue is vital. Although the dECM may contain an intact vascular network, the decellularization process induces significant structural changes in this vascular network [40]. In a comparative study between decellularization protocols of SDS with DNase or SDS with Triton X-100, the SDS with Triton X-100 treatment of porcine kidneys was shown to better preserve microvascular structures such as the glomeruli [41]. This treatment also allowed for improved vascular function when re-endothelialized and perfused with blood in vitro. Figure 2 shows a comparison of the native kidney, SDS with DNase treated dECM, and Triton X-100 and SDS treated dECM decellularization methods, the vascular corrosion casts of each, and the vascular microarchitecture of each scaffold. The dECM treated with Triton X-100 and SDS showed similar vascular structures to those in the native kidney while the dECM treated with DNase and SDS showed defect areas that are marked by arrows in the figure. 

It has been observed that in addition to chemical/ biochemical agents used, other factors such as flow rate and temperature must also be optimized for decellularization methods. Furthermore, more research is needed to determine whether dECM from other animals, such as porcine dECM, can be used in human clinical applications. Although the biocompatibility and hemocompatibility of porcine dECM have been demonstrated with human cells, further investigation is needed to determine the most optimum source of dECM. 

The most challenging aspect of using dECM in TE is the recellularization process. Particularly, the re-endothelialization of the kidney for a complete, functioning vascular network presents many challenges. An advantage of dECM approaches, however, is that in addition to matrix-to-cell signaling, cell-to-matrix signaling can also occur in which the original dECM is gradually remodeled or replaced by cell-derived, autologous ECM, making the application of xenografts in TE more feasible. It has been shown that pluripotent mouse embryonic stem cells seeded into rat kidney dECM endothelialized and remodeled the rat basement membranes, suggesting successful matrix-to-cell, cell-to-cell, and cell-to matrix communication [42]. The cells lined the blood vessels of the glomeruli and produced endothelial lineage markers while the rat dECM basement membrane eventually incorporated mouse laminin and type IV collagen. 

**Figure 3:**
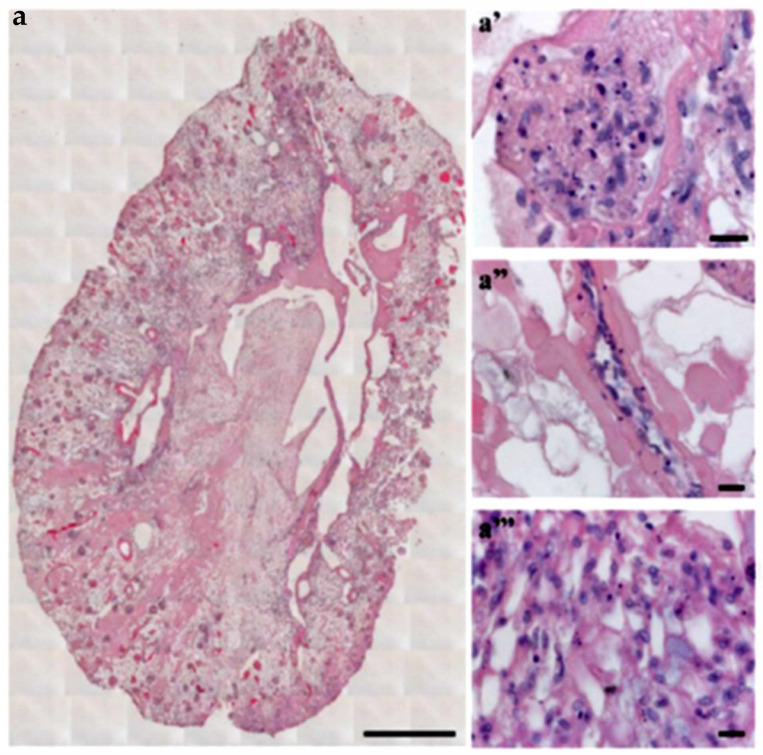
Decellularized kidney scaffold obtained from adult male rat reseeded with iPSC-derived endothelial cells. (**a**) Cross-section of repopulated kidney showing homogeneous distribution of iPSC-derived ECs into glomeruli and vascular structures. Scale bar 1 cm. (**a**′–**a**‴) Images showing iPSC-derived ECs localization into glomerulus (**a**′), vascular network (**a**″), and peritubular capillaries (**a**‴). Scale bar 20 μm. (Reproduced with permission from Springer Nature under Creative Commons License, adapted from reference [43].)

The recellularization methodologies used for kidney dECM are critical for achieving successful cell distribution and differentiation. In a recent study, it was shown that the infusion of human induced pluripotent stem cell (hiPSC)-derived endothelial cells from the renal artery and vein of rat kidney dECM resulted in the partial repopulation of all vasculature components [43]. As shown in Figure 3, endothelial cell localization in different parts of the kidney was observed. Furthermore, the glomerular capillaries contained fenestrated endothelia while the small arterioles contained strings of endothelial cells in a single layer covering the inner vessel wall. Overall, the glomeruli were successfully repopulated with hiPSC-derived endothelial cells forming a continuous network. This study achieved an 89% repopulation of all glomeruli, indicating that further improved methods for achieving more uniform and complete cell distribution are needed. Many efforts to remodel the dECM before recellularization in order to improve scaffold repopulation have been made. The preloading of both rat and human kidney dECM with VEGF and angiopoietin-1 allowed for successful re-endothelialization of the entire kidney vasculature with hiPSC-derived endothelial cells [44]. Normally, pericytes produce VEGF and angiopoietin-1 in the developing kidney to promote endothelial cell differentiation and vascularization, so the preloading of these growth factors in the dECM could serve as a replacement for pericytes which typically make the re-endothelialization of small capillaries difficult due to their large size. The growth factors were shown to promote hiPSC adherence and survival within the dECM scaffold as well as the eventual expression of endothelial differentiation markers. However, some obstructed afferent arterioles were still observed, highlighting the challenges of repopulating the intricate kidney microvasculature. This preloading of growth factors along with an arteriovenous delivery system of hiPSCs did result in improved vascular functioning of the graft. Thus, the simultaneous arteriovenous delivery systems have proven to be effective in kidney dECM recellularization. Other attempts at remodeling the kidney dECM before recellularization include glutaraldehyde cross linking [45] and heparin immobilization [46]. Glutaraldehyde cross-linking of dECM increases the overall mechanical strength and structural integrity of the dECM. When implanted in mice, these cross-linked scaffolds showed good histocompatibility, and when seeded with fibroblasts, they showed good cytocompatibility. The immobilization of heparin to collagen on kidney dECM using collagen-binding peptide provides significantly improved conditions for neovascularization by preventing thrombosis and promoting re-endothelialization. Normally, exposed collagen in dECM promotes coagulation of perfused blood, so the immobilization of heparin to exposed collagen was one attempt at overcoming these thrombogenic tendencies of kidney dECMs. Heparin immobilization ultimately showed an increased retention rate and attachment strength of endothelial cells as well as decreased thrombogenicity upon whole-blood reperfusion. The practice of remodeling the kidney dECM scaffold holds great promise for eventual whole organ TE applications. 

Another use for kidney dECM scaffolds is the deconstruction of dECM architecture and the construction of dECM-derived hydrogels. The most common engineered scaffolds made from natural biomaterials incorporate kidney dECM hydrogels [47]. Hydrogels derived from kidney dECM have the advantage of preserving the native ECM protein ratios of the kidney while providing highly tunable mechanical stiffness through gelation [48]. Although dECM scaffolds with preserved kidney structures provide structural guidance for cell seeding, especially with vascularization, these scaffolds tend to lack structural flexibility and can often restrict cell migration and complete cellular repopulation. By further processing kidney dECM into hydrogels through homogenization, researchers have been able to preserve the proper biochemical cues of the scaffolds while creating more suitable mechanical robustness. dECM hydrogels were shown to support growth of conditionally immortalized human glomerular endothelial cells better than hydrogels of purified collagen I [49]. Human kidney peritubular microvascular endothelial cells cultured in decellularized kidney cortex ECM-derived hydrogels on a planar surface maintained a quiescent state as opposed to when cultured on a collagen I hydrogel [50]. Compared to collagen I hydrogels, these cells cultured on kidney dECM hydrogels exhibited reduced CD31 expression with less even distribution. Various dECM modifications, such as dECM scaffolds with added hypoxia-inducible factor-1a, have also been used in successful kidney cell culture [51]. While dECM hydrogels are widely used, a significant limitation of this approach is that ECM composition greatly varies between different kidney structures, so different hydrogels might need to be constructed for different sections of the kidney. Ultimately, a hydrogel scaffold, of synthetic or natural materials, with similar composition and potential for kidney cell support to dECM hydrogels should be developed to eliminate the need for donor kidneys. 

Although decellularization of kidneys followed by recellularization or hydrogel formation has been investigated for many years with procedural improvements and novel techniques emerging constantly, this approach has inherent limitations that may prevent widespread application. The ultimate goal of TE is to overcome the need for donor organs, and dECM approaches will always require donor organs from human or animal sources as well as large quantities of cells. However, due to the complex architecture of the kidney and particularly the highly specialized kidney vasculature with no other effective methods for developing those architectures de novo, dECM approaches might be the most effective means of engineering a whole kidney. 

### 3.2. Vascular Corrosion Casts

**Figure 4:**
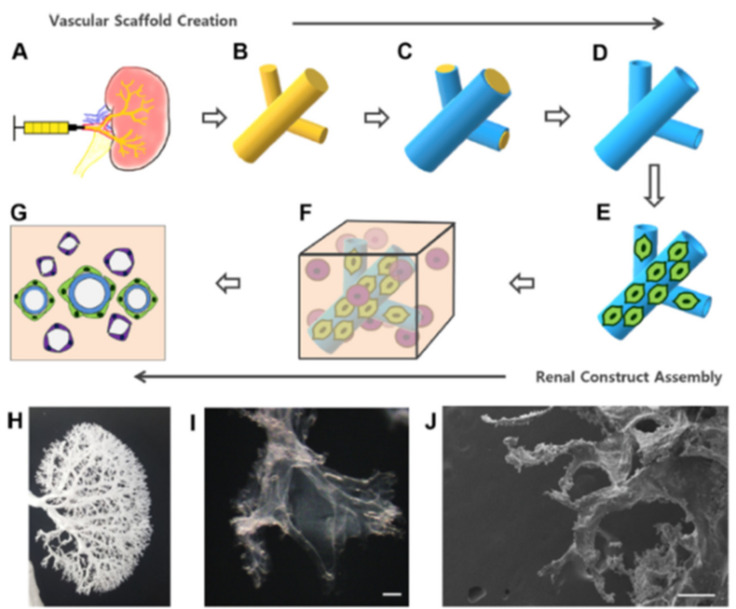
Fabrication process of a pre-vascularized biomimetic scaffold and 3D renal construct. (**A**) PCL solution was perfused into the renal artery. (**B**) The kidney tissue was digested with NaOH, resulting in a PCL vascular corrosion cast. (**C**) The PCL cast was dip coated with collagen. (**D**) The PCL cast was removed with acetone, leaving a hollow collagen vascular scaffold. (**E**) The MS1 cells were coated onto the vascular scaffold. (**F**) The complete renal 3D construct was made with MS1-coated vascular scaffold in collagen hydrogel mixed with renal cells. (**G**) Renal cells self-assemble into tubule-like structures over time. (**H**) Gross image of a PCL vascular corrosion cast. (**I**) Microscopic image of a collagen vascular scaffold. (**J**) SEM image of a collagen vascular scaffold. Scale bar = 200 µm. (Reproduced with permission from Elsevier, adapted from reference [53].)

Typically, vascular corrosion casts are used to visualize the vasculature of a scaffold, such as when examining a dECM scaffold to ensure the vasculature has been preserved following decellularization. However, Huling and co-workers recently showed that vascular corrosion casts can be utilized as a means for developing a biomimetic vascular scaffold for potential regenerative medicine strategies or even whole-kidney TE applications [52,53]. Similar to the dECM approaches where the entire kidney ECM structure is preserved and recellularized, this technique uses a vascular corrosion cast to provide a whole-organ structure on which cells can be seeded. The general procedure for developing these pre-vascularized scaffolds is shown in Figure 4. The cast is made by perfusing a polyε-caprolactone (PCL) solution into rat kidneys through the renal artery which is then followed by complete tissue digestion. The remaining PCL cast mimics the natural vascular structure of the kidney. Results showed that the vessels in the kidney cortex and glomerular capillaries remained intact in the PCL vascular casts. By coating the PCL cast with rat type I collagen and removing the PCL by dissolving in acetone, a hollow, biomimetic collagen-based scaffold that mimics the native kidney vasculature remained. The collagen scaffold is soaked in a crosslinking solution of EDC and NHS. Mile Sven-1 (MS1) endothelial cells were then coated on the collagen scaffold as a pre-vascularization technique. These endothelial cell-coated scaffolds were embedded in a type I collagen 3D hydrogel, and a heterogeneous population of human renal cells and renal growth medium were added to the hydrogel. Scaffolds were incubated in the cell-loaded hydrogel before implantation into rats with renal cortical defects. The cell-loaded scaffolds were split into smaller parts and placed within these renal defects where they were secured in place with fibrin glue. 

The implanted partial renal tissue constructs showed strong evidence of anastomosis to host renal vasculature after two weeks. The vascular channels of the scaffolds were filled with nucleated cells and red blood cells from the host, suggesting successful blood flow between the rat kidney and the implanted scaffold that allowed for enhanced neovascularization and renal cell survival. Unexpectedly, the MS1 cells that had been used to pre-vascularize the collagen scaffolds did not remain attached to the scaffolds but rather scattered throughout the constructs to form microvascular channels with host-derived endothelial cells. Although the MS1 cells did not remain attached to the scaffolds, it was still demonstrated that the presence of the scaffold was necessary for MS1 cell survival, possibly due to the increased blood and oxygen supply through the prefabricated vessels. Future studies could attempt seeding the endothelial cells on the inside of the vascular corrosion casts to prevent migration away from the scaffold, but the small size of the kidney capillaries might introduce obstacles to a direct cell perfusion method. Ultimately, this study served as a proof of concept that partial renal implantation, and perhaps even whole-kidney TE, could someday be achieved with cell-seeded vascular corrosion casts, but many procedural adjustments must still be made. These in vivo scaffolds showed low efficiency of renal tubule structure formation as compared to in vitro cultures, suggesting the need for new techniques for improving integration into the host tissue. 

## 4. Kidney Organoids—The Bottom-Up Approach

Development of stem cell-derived miniature organoids can be thought of as a bottom-up approach to TE. The process starts with individual stem cells that can be programmed to differentiate towards a renal lineage and eventually develop into kidney structures through self-assembly, similar to in vivo counterparts in human fetal development. Kidney organoids show great promise for future TE and regenerative medicine applications, and organoids cultured in microfluidic systems show potential for improved in vitro vascularization that will allow for an increase in the size of organoids for eventual clinical applications. 

The development of in vitro kidney organoids is possible because of the extensive research that has gone into understanding the molecular and cellular basis of embryonic kidney development. Furthermore, the ability to reprogram terminally differentiated adult cells back to induced pluripotent stem cells (iPSCs) has completely transformed the field of regenerative medicine and enabled scientists to obtain large amounts of autologous hPSCs without the controversial use of human embryonic stem cells (ESCs) [54]. The ability to use iPSCs for patient-derived, tissue-specific organoids has sparked tremendous growth in the field of TE. By emulating the molecular signaling processes of embryonic kidney development, scientists can now induce hiPSCs to form complex kidney structures in vitro which eventually can form kidney organoids with functioning nephrons [55]. The comparison between kidney organoids and human fetal kidneys has shown that the organoids do indeed closely mimic the natural embryonic development of human kidneys, although the organoids remain as rather immature forms [56]. Several review articles have been published that highlight the recent developments and future applications of kidney organoids [57,58].

hPSC-derived nephron progenitor cells (NPCs) are multipotent stem cells that can develop into nephron-like structures comparable to their in vivo embryonic counterparts [59]. NPCs undergo mesenchymal-to-epithelial transition upon Wnt stimulation, which is the protocol used for most formation of kidney organoids [60]. Interestingly, iPSCs from patients undergoing hemodialysis due to a disease also can differentiate into NPCs with similar efficiency as iPSCs from healthy donors, indicating promising future applications for TE with the autologous cells of patients with kidney diseases. NPCs are, however, separate from the kidney stroma and vasculature, and non-nephron cells are also a necessary component of kidney organoids as they help establish a multi-compartment environment that can support vascularization, particularly in glomerular and tubule-interstitial structures. In their work, Morizane et al. reported a high-efficiency protocol for generating NPCs and kidney organoids from multiple lines of both hESCs and hiPSCs [61]. It has been proposed by Low and co-workers that a subset of NPCs may serve as a source of renal vasculature as well [62]. This subset of NPCs displays a vascular progenitor-like property and eventually differentiates into more mature endothelial cells. They reported in vitro vascularization of a de novo vascular network in organoids derived from a subset of NPCs. Through Wnt signaling, the proportion of proximal and distal segments were carefully controlled, resulting in glomerular podocytes producing sufficient VEGFA for a defined vascular network. The procedure did not require exogenous VEGFA, and when VEGFA signaling was disrupted by VEGF receptor inhibitors, the vascular network was notably diminished while the nephrons remained the same. When implanted beneath the renal capsule of immunocompromised mice, an increase in organoid size, maturity, and vascularization was observed. Particularly, podocytes developed elaborate cellular processes and glomeruli developed a more mature architecture with distinct capillary tufts perfused by red blood cells. A fenestrated endothelium and glomerular basement-like membrane also developed, contributing to the establishment of a glomerular filtration barrier capable of performing preliminary filtration and reabsorption. Although this study successfully induced in vitro vascularization of organoids, the eventual in vivo environment was nonetheless required for maturation of a functioning vascular network.

To prepare fully functional kidney organoids, a wide array of differentiation protocols has been developed, all of which possess different benefits and levels of complexity. The differentiation protocols reported by Takasato et al. include both hESC-derived and hiPSC-derived organoids that successfully model human nephrogenesis [63,64]. The differentiation protocols often involve alternating usage of CHIR, a small molecule Wnt activator, FGF9 (a fibroblast growth factor), and activin, a TGFβ activator followed by a period without any growth factors. Recently, the Takasato protocol reports the formation of organoids with all anticipated kidney cell types, segmented nephrons, a renal interstitium, and an extensive endothelial network [65]. NPCs developed within nine days and organoids developed within twelve days. The protocol enables mass production of 3D kidney organoids by using 96-well, ultra-low-attachment plates. Guidelines for adjusting the protocol for different lines of hPSCs will allow for greater quality control between batches. While the nephrons were well characterized, cells in the interstitial space, including pericytes, endothelial cells, smooth muscle cells, and fibroblasts, were not. Another simple and inexpensive bioreactor-based method for growing kidney organoids in bulk from hiPSCs was reported by Przepiorski and co-workers but further research is required to minimize the fibrotic tendencies of these organoids [66]. The differentiation protocol included the use of CHIR and knockout serum replacement, but no FGF9, and spinner-flask bioreactors were used to improve oxygen and nutrient perfusion. A 21-day protocol using high-throughput screening for kidney organoid differentiation performed entirely by liquid-handling robots has also been proposed, but organoids produced by these extremely efficient methods are likely more applicable for use in toxicity and disease screening rather than regenerative medicine applications due to their small size and relatively immature nephrons [67]. Another cost-effective method for developing kidney micro-organoids resulted in 6–10 nephrons per organoid and all anticipated renal cell types [68]. The differentiation protocol involves activation with CHIR, FGF9, and heparin in matrigel-coated monolayer cultures under low speed swirling using suspension culture approach. Although relatively immature, the nephrons were surrounded by stromal and endothelial cell types. These could serve as a good source of hPSC-derived kidney cells because the suspension culture approach produces up to four times as many cells as static culture. 

In a recent study, Taguchi and co-workers showed that endothelial cells are not always required for the initial branching morphogenesis of in vitro kidney organoids [69]. Many reported kidney organoids contain very few endothelial cells [70,71], and even when kidney organoids do contain endothelial cells, the eventual vascular networks found within in vivo organoids are derived entirely from the host. Furthermore, most glomeruli within in vitro organoids are avascular, and vascularization is not achieved without in vivo transplantation. Although in vivo vascularization would have poor commercial and industry applications, in vitro vascularization of organoids is proving to be a significant challenge [72]. In some in vivo vascularization studies, the vasculature that developed after implantation was entirely derived from the host, and in other studies the organoids themselves contributed cells to vascularization. Ideally, a kidney organoid should be able to contribute as much as possible to the eventual vasculature since a pre-vascularized organoid could allow for much larger sizes before implantation. Early studies of relatively simple kidney organoids implanted in rats showed early vascularization of glomeruli and the differentiation of nephrons with transport capabilities [73]. Embryonic stem cell (ESC)-derived organoids co-cultured with embryonic spinal cord segments transplanted beneath the kidney capsule of immunodeficient mice developed vascularized glomeruli that connected to host circulation and contained red blood cells [74]. Co-culture with spinal cords was used instead of the more common Wnt stimulation differentiation procedure, and recent organoid protocols have used iPSCs instead of ESCs. Thus, in vitro organoids that possess a well-developed network of endothelial cells before implantation, are generally well vascularized and connected to the host circulation after implantation. 

Several studies have reported a combination of both host-derived and organoid-derived vasculature upon transplantation. hPSC-derived kidney progenitors subcutaneously implanted in immunodeficient mice formed organ-like masses exhibiting functioning nephrons and vascularization [75]. The glomeruli were mature and perfused, containing human capillaries and podocytes, confirming the presence of hPSC-derived vasculature. Dextran uptake from glomerular filtrate was observed, and implanted kidney progenitors survived for over three months. In an attempt to evaluate the organoid versus host contribution to endothelial cells for vascularization after transplantation, Murakami et al. found that the majority of the renal vasculature in their organoids upon transplantation came from donor-derived rather than host-derived endothelial cells [76]. The organoids were developed from mouse embryonic kidney cells and implanted under the kidney capsule of immunodeficient mice. Although the organoid cells were the primary source of vasculature, the in vivo implantation was still necessary as organoids only developed minor vasculature in vitro. The donor cell contribution to the vasculature came from a newly identified set of renal endothelial precursors present in the embryonic mouse kidney that were able to reorganize the arteriolar structure more efficiently than the host-derived endothelial cells. They proposed that this new set of endothelial precursors could be an ideal target for induction from ESCs or hiPSCs for future organoid vascularization applications. 

Although less common than in situ organoid vascularization, the transplantation of organoids for host-derived vasculature at sites other than the kidney, or ex situ, shows promising applications as well. In ex situ transplantation, the host body is used as a bioreactor in a secondary location prior to re-transplantation at the target site with a second surgery. One such experiment with kidney organoid vascularization was the growth of a kidney-in-a-lymph node as an in vivo pre-vascularization approach using the lymph node as a secondary location [77]. This study implanted human kidney progenitors from human fetal kidneys into mouse lymph nodes. Single-cell suspensions failed to differentiate within the lymph node, but kidney progenitors allowed to first differentiate into organoids could subsequently mature in the lymph node’s in vivo environment. Those in vivo organoids exhibited excretory, homeostatic, and endocrine functions with a significant amount of host-derived vasculature. The lymph node has been shown to function as an effective bioreactor for many types of engrafted cells [78]. Previous studies have shown that even normal somatic cells derived from autologous tissues may cause immunological responses, so it has been suggested that the lymph node may be a potent transplantation region for reprogrammed somatic cells that can then be utilized for tissue regeneration. The structure of the lymph node is such that it provides immediate availability to endothelial capillaries and therefore critical nutrients and growth factors from the blood. Furthermore, lymph nodes contain fibroblastic reticular cells and other stromal cells that secrete chemokines to enhance cell recruitment, growth, expansion and survival [79,80]. Thus, it may be hypothesized that lymph nodes may be utilized for potential kidney tissue regeneration as well. 

Various 3D microenvironments for kidney organoid culture that do not involve implantation into a host animal have also been reported. For example, Garreta et al. proposed chick chorioallantoic membrane (CAM) as a suitable environment for generating renal vesicles, nephron structures, and vasculature in kidney organoids from hPSCs [81]. CAM is a naturally highly vascularized extraembryonic tissue and provides a soft in vivo microenvironment that promotes organoid vascularization. They compared the organoids grown on CAM to organoids grown on 3D hydrogels fabricated from functionalized polyacrylamide of tunable stiffness designed to mimic the CAM mechanical properties in vitro. Organoids grown on both of the soft 3D microenvironments were compared to organoids grown in rigid conditions. Results showed that organoids cultured in rigid conditions possessed vascular endothelial cells surrounding the nephron structures but lacked a vascular network. However, blood vessels from the CAM invaded organoids, interacting with the glomerular structures, and chick blood was able to circulate through the organoids. The kidney organoid structures were also more mature than organoids not cultured on CAM, with enlarged BowmaN’s capsules and aligned podocyte-like cells. Organoids cultured on the engineered soft 3D polyacrylamide hydrogels showed better nephron structures and vascularization markers than organoids cultured on rigid conditions. However, these organoids still had to be eventually implanted onto CAM for vascularization to occur. RNA-sequencing analysis revealed that organoids cultured on these soft 3D microenvironments at day 16 transcriptionally matched human second-trimester fetal kidneys, validated by markers of nephron progenitors such as SIX2 and PAX2, markers of proximal tubules such as SLC3A1, and markers of glomeruli such as NPHS1 and PODXL via qPCR.

Kidney organoids hold incredible potential for engineering immunocompatible transplantable kidneys from a patient’s autologous cells, but their applications are far from reaching the clinical level. Problems include the reproducibility of the differentiation protocols, differences between hPSC lines, and the limited degree of maturity and functionality that can be obtained due to a lack of blood supply. Although cellular heterogeneity is the main feature that gives kidney organoids their primary advantage, it is also one of the most challenging aspects of kidney organoid-based approaches. With over twenty cell types that must be organized in precise, intricate structures, the kidney organoid is an extreme challenge from a manufacturing perspective. There is great variability between different batches of human kidney organoids [82]. Because kidney organoids are some of the most complex, the challenge of reproducibility is substantial. A thorough evaluation of variability in kidney organoids showed significant transcriptional variation between batches, with varying levels of maturation and off-target, or non-kidney, populations [82]. 

A potential limitation to applying kidney organoids in a clinical setting is similar to risks associated with other stem cell therapies. Stem cells have the risk of forming off-target populations. Certain kidney organoids may have fibrotic tendencies. Furthermore, some sub-capsular grafts will exhibit inosculation to the host vasculature, but the collecting duct system does not connect to the host in order to facilitate urine outflow. Methods for the most effective inosculation of kidney organoids with host vasculature should be further explored to allow for the growth of organoids to more clinically relevant sizes in vivo [83]. Although kidney organoids are far from reaching clinical translation, these obstacles are by no means insurmountable. The speed at which this field progresses is promising, and the in vitro culture of kidney organoids under flow in microphysiological systems could serve as an alternative route to organoid vascularization. 

### Microphysiological Systems

**Figure 5:**
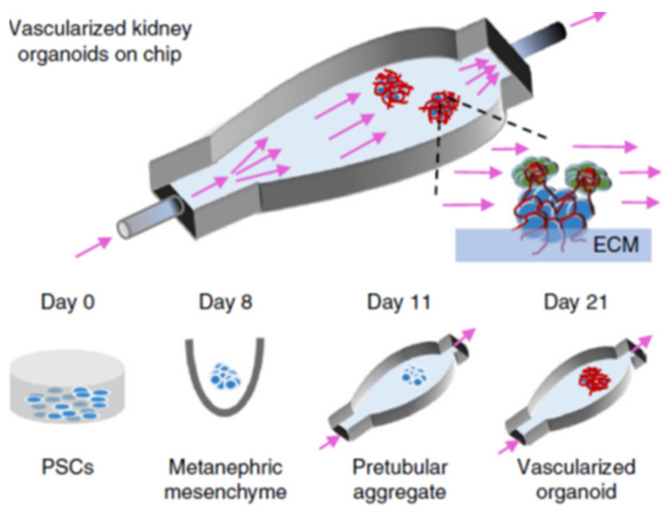
Formation of renal organoids placed on engineered ECM, in a perfusable millifluidic chip, under controlled fluid shear stress. Differentiation days and culture conditions are indicated in the middle and bottom parts of the panel. Organoids not drawn to scale. Reproduced with permission from Nature (adapted from reference [86]).

Microphysiological systems, or organ-on-a-chip models, are essentially finely tuned cellular microenvironments that have biological applications in pharmacology, toxicology, and disease modeling. The ability to develop in vitro microscale technologies that regulate stem cell differentiation and model microvasculature in 3D organoids has promising applications for tissue engineering [84]. As discussed in the section on kidney organoids, inducing the vascularization of kidney organoids in vitro is challenging. While a majority of kidney organoid vascularization approaches involve in vivo implantation with host-mediated vasculogenesis and angiogenesis, microphysiological systems have been used as in vitro organoid vascularization approaches. Microfluidic design principles can be utilized to create functional vascular networks with tunable properties tailored to specific organs; the types of vascular cells, flow dynamics, lumen diameter, and vessel branching architecture can be precisely controlled [84]. Photolithographic techniques allow for unique 3D micropatterns that can mimic organ vasculature branching [85]. Ultimately, the importance of microfluidics in developing organ structure and vasculature is highlighted in microphysiological systems [86]. Vascular perfusion is needed to guide cell migration and differentiation, polarize cells, and regulate filtration and transport functions [87]. Flow-enhanced vascularization and maturation of kidney organoids on 3D-printed microfluidic chips has been the most promising method of in vitro organoid vascularization to date. The microfluidic chip consists of a gelatin-fibrin ECM coating on a 3D-printed chip which allows fluid flow between the ECM and chip surface. Flow over the top surface of organoids with high fluidic shear stress and co-culture with human endothelial cells resulted in vascular networks with significantly increased junctional density and vessel length. The percentage of the vascular surface area in contact with organoid tubules was threefold for high fluidic shear stress compared to low fluidic shear stress. Figure 5 shows the procedure for development of renal organoids that are placed on an engineered ECM, contained within a perfusable millifluidic chip, and exposed to different amounts of fluidic shear stress. Results showed that significantly enhanced vascular networks were observed with increasing fluidic shear stress. Glomerular structures were also vascularized under high fluidic shear stress while nearly avascular structures formed in low fluidic shear stress or static culture. With higher vascularization levels, more advanced morphogenesis of tubular epithelial cells was also observed. 

In addition to the vascularization of organoids, microfluidics has the potential to further optimize the organoid differentiation protocol and mass produce highly controlled organoids in vitro. Glass et al. recently optimized a microbioreactor procedure for generating kidney organoids from hPSCs under perfusion [88]. The microfluidic device was fabricated by soft lithography on silicon wafers using layers of polydimethylsiloxane (PDMS), and organoids were developed from human ESCs. Growing the organoids within this finely tuned microphysiological system allowed for improved control over the complex differentiation process as well as the ability to closely analyze and better understand the differentiation process through the number of experimental protocols. 

Rather than attempting the immense task of engineering a whole kidney, many groups have focused on smaller-scale engineering of individual kidney structures or isolated kidney functions within microphysiological systems for both in vitro and in vivo applications. These smaller-scale systems follow the principles of renal assist devices (RADs), or extracorporeal devices made of cells that can be used to enhance kidney function and used in combination with dialysis [89]. Ideally, these devices would be implantable and augment the functions of one or more failing kidney structures. Additionally, the development of these individual kidney structures and functions allows for a better understanding of kidney TE with eventual whole organ TE applications. Petrosyan et al. demonstrated this concept with their glomerulus-on-a-chip using human podocytes and glomerular endothelial cells under bidirectional flow [90]. Seeded podocytes formed slit diaphragms, endothelial cells formed capillary-like structures, and the deposited basement membrane contained specific glomerular extracellular proteins. The glomerulus-on-a-chip was highly reproducible and maintained these kidney-specific phenotypes and functions for at least a month in vitro. 

Another example of an isolated kidney function on an in vitro microfluidic chip is the reconstruction of the human renal vascular-tubular interactions [91]. Using type I collagen hydrogel membrane and overlapping fabricated vascular or tubular lumens containing human kidney peritubular microvascular endothelial cells (HKMECs) or human kidney epithelial cells, a microphysiological system was designed that demonstrated renal-specific functions such as selective reabsorption of glucose and albumin. The vascular and tubular microfluidic channels were fabricated using photolithographic, soft lithographic, and injection molding techniques, and continuous exposure to flow resulted in a remodeled collagen membrane that mimicked the native kidney matrix and basement membrane, flow-directed alignment of endothelial cells, and intact cellular junctions as confirmed by VE-cadherin staining. Human fetal kidney pericytes incorporated into the collagen matrix were shown to grow processes along the surfaces of both epithelial and endothelial channels. This ability to recreate such vascular-tubular interactions of the kidney within a highly tunable microfluidic system could potentially lead to clinical applications in the form of RADs or significant advances in the functioning of kidney organoids. 

**Figure 6:**
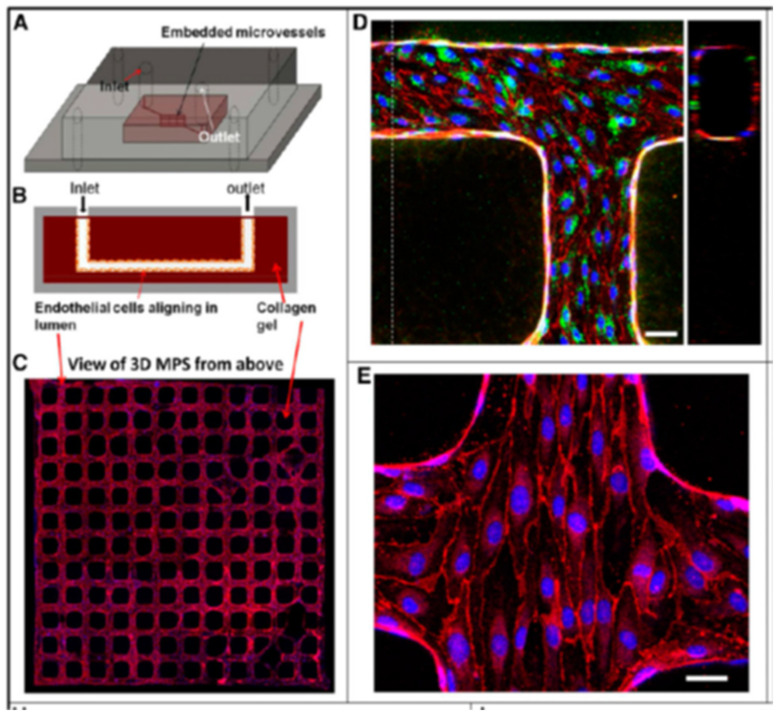
Schematic illustration of 3D the microphysiological system: (**A**) 3D views; (**B**) cross-sections. (**C**) Formation of kidney microvessel networks. Red indicates CD31, and blue indicates nuclei. (**D**) Confocal microscopy image of engineered human kidney microvessel. Red indicates CD31, green indicates vWF,. Scale bar, 50 mm. (**E**) Confocal image of human kidney microvessels at a junction of the network. Red indicates VE cadherin, and blue indicates nuclei. Scale bar, 25 mm. (Reproduced with permission from the American Society of Nephrology. Adapted from Reference [92].)

Microfluidic devices have been used particularly to examine and construct the kidney microvasculature. It has been shown that kidney peritubular microvessels are highly susceptible to injury and show limited regenerative capacity. The incorporation of HKMECs in various flow-directed microphysiological systems has enabled the construction of 3D kidney microvascualture networks and a better understanding of the roles flow and pressure play on endothelial cell heterogeneity. The flow-directed, three-dimensional kidney microvasculature model described by Ligresti et al. showed that the use of HKMECs over HUVECs (human umbilical vein-derived endothelial cells) resulted in kidney-specific phenotypes [92], as shown in Figure 6. The microvascular networks were also made in collagen gel using lithographic processes. HKMECs from both adult and fetal kidneys under flow formed a thin endothelial membrane with fenestral diaphragms similar to native kidney structure and a functional permeability barrier. The endothelium was found to express CD31 and VE cadherin at regions of cell–cell contact, and a basement membrane consisting mostly of collagen IV was deposited along the microvessel walls by HKMECs. The results of immunohistochemistry studies of the microvessel junctions showed strong expression of F-actin and plasmalemma protein PV1. The fact that PV1 was so strongly expressed throughout the endothelium when HKMECs were used suggests that HKMECs are necessary to produce the proteins needed to form the diaphragms that bridge the endothelial fenestrae. Because the HKMEC microvessels possessed more fenestrae, the vessel permeability was also higher compared to those formed in the presence of HUVEC cells. Proper junctions were formed at cell–cell contacts, and numerous closed fenestrae throughout the microvessel walls were seen. The average size of these fenestrae was very similar to that of fenestrae found in kidney microvessels in vivo. Although these miniature kidney microvessel models represent a very small portion of the engineered kidney, the ability to recreate very kidney-specific phenotypes in vitro could potentially be applied to larger-scale kidney TE. 

## 5. Conclusions and Future Directions

We have summarized the four major kidney tissue engineering methodologies discussed in this review in Table 1. Vascularization is one of the major challenges that remains in the engineering of large organs such as the kidney that require an elaborate network of arteries, veins, and capillaries to prevent necrosis in the innermost part of the tissue. The demand for vascularization in renal TE particularly remains higher than ever. An alternative vascularization strategy that attempts to overcome the lack of organ-specificity in the inosculation of pre-vascularized tissues is in situ angiogenic remodeling. 

With angiogenic remodeling, host blood vessels grow into the engrafted engineered tissues to allow for a higher degree of organ-specificity in the regenerated vascular networks. However, the location of the kidney in the body makes in situ angiogenic remodeling a great challenge, so improvements to pre-vascularization strategies should be a main focus of kidney TE. Many scaffolds and cell types have been reported for pre-vascularization techniques, but more kidney-specific applications are needed. Other techniques such as photolithography have been used for TE, but there are limitations due to high costs, high temperature requirements, exposure of cells to UV light (causing DNA damage), and the etching process itself that may require toxic solvents. Moreover, photolithography has very little control over surface chemistry, particularly when not confined to planar surfaces. One area of technology that has seen great progress in the field of TE vascularization but has yet to be applied to whole-kidney TE is 3D bioprinting. In contrast to conventional tissue engineering methodologies, progress has been made in 3D and 4D bioprinting systems, which may allow for more accurate positioning and directionality between the individual structural elements of specific tissue but, as yet, applications for organs as complex as the kidney remain limited. With the improvement of 3D bioprinting techniques and kidney-specific bioinks, as well as 3D bioprinting approaches to graft vascularization, a more precise and reproducible method for constructing kidney organoids or even whole organs could be realized. 

A majority of approaches to kidney TE currently employ dECM scaffolds, which can then be repopulated with the patient’s autologous cells. Decellularized rat, porcine, and human kidneys have been successfully seeded with epithelial and endothelial cells to produce grafts that produce rudimentary urine both in vitro and in vivo. Infusion of hiPSC-derived endothelial cells through the renal artery and vein of rat kidney dECM was found to restore vasculature components. Although dECM helps maintain kidney structure, vascular integrity, and biocompatibility, it possesses significantly reduced mechanical strength, and it is difficult to entirely repopulate with appropriately differentiated cells, and moreover does not eliminate the need for donor organs. Methods of improving dECM mechanical strength and cell repopulation should certainly be explored in the meantime, but alternative bottom-up strategies should be the ultimate goal of kidney TE. 

Two relatively new approaches that show promise for regenerating kidneys are the use of organ-on-a-chip models and stem cell-derived miniature 3D organoids. While kidney-on-a-chip models are useful for modeling vasculature and growing isolated kidney structures, such as a glomerulus-on-a-chip, such organ-on-a-chip models are still far from functioning in replacement and regrowth of damaged kidney tissue in vivo. 3D kidney organoids made from directed differentiation of PSCs contain multiple cell types found in the human kidney and possess impressive hierarchical levels of organization with nephron-like structures and vascularization. Cost-effective methods have been developed for growing kidney organoids in bulk from hiPSCs. The asynchronous mixing of kidney progenitor cells at distinct stages of differentiation has been shown to promote nephrogenesis, vascularization, and eventual inosculation of kidney organoids upon transplantation into mice. While the advances seen with kidney organoids hold great promise, they are still too functionally immature, physically small, and under-vascularized to have direct clinical applications. Furthermore, there is great variability between different batches of human kidney organoids, so more reproducible differentiation protocols must be developed. As things stand today, it appears that the merging of the organ-on-a-chip and 3D organoid techniques by culturing kidney organoids under flow on microfluidic chips may hold the most promise for developing functioning, vascularized grafts for TE applications. 

Thus far, the most effective attempts at mimicking the kidney vasculature have been through the use of flow-enhanced kidney organoids, re-endothelialized dECMs, or vascular corrosion casts to create a collagen-based biomimetic scaffold that preserves the natural kidney vasculature. In addition to kidney dECM or stem cell-derived organoid approaches, a wide variety of synthetic and natural biomaterials have been used to construct scaffolds that promote vascularization and nephrogenesis of engineered kidney tissue, but clinical applications remain limited. The combination of the top-down and bottom-up approaches discussed in this review with novel biomaterials designed specifically for kidney tissue growth, differentiation, and functioning will continue to improve the field of renal TE and progress closer to the realization of vascularized, functioning kidney grafts to replace organ transplants in clinical applications for chronic kidney disease.

## Figures and Tables

**Table 1:** Comparison of developments and limitations of top-down and bottom-up approaches discussed in this review.

Top-Down Approaches	Bottom-Up Approaches
**dECM** Maintains native kidney architecture & biochemical cues. Problems with recellularization & mechanical strength Requires donor kidney	**Organoids** Only requires a small amount of patient-derived stem cells. Difficulties with reproducibility Limited to small sizes due to lack of complete vascularization & implantation sites.
**Vascular Corrosion Casts** Maintains native kidney vasculature architecture Can be made from a variety of materials Issues with cell-seeding and integration with host-tissue	**Microphysiological Systems** Improved *in vitro* vascularization of organoids due to microfluidics. Precise control over chip dimensions and flow dynamics. Limited to small sizes & mostly 2D applications.

## Data Availability

Not applicable.
